# Effectiveness of extracorporeal shock wave for post-stroke shoulder-hand syndrome: A protocol for systematic review and meta analysis

**DOI:** 10.1097/MD.0000000000020664

**Published:** 2020-07-02

**Authors:** Tian-shu Wang, Shou-feng Wang, Wei-dong Song, Zhao-chen Tang, Yu Zhao, Ken Lee

**Affiliations:** a Second Ward of Orthopedics Department; b First Ward of Orthopedics Department, First Affiliated Hospital of Jiamusi University; c Department of Orthopedics, Second Affiliated Hospital of Mudanjiang Medical University, Mudanjiang; d School of Clinical Medicine, Jiamusi University, Jiamusi; e Department of Orthopedics, Huludao Central Hospital, Huludao, China; f School of Social and Community Medicine, University of Bristol, Bristol, UK.

**Keywords:** effectiveness, extracorporeal shock wave, post-stroke shoulder-hand syndrome, safety

## Abstract

**Background:**

Post-stroke shoulder-hand syndrome (PSSHS) is one of the most common sequelae in patients with stroke. Previous studies have reported that extracorporeal shock wave (EPSW) has been used to treat this condition effectively. However, its conclusions are still inconsistent. Therefore, this study will provide evidence to systematically assess the effectiveness and safety of EPSW for the treatment of PSSHS.

**Methods:**

We will comprehensively search relevant randomized controlled trials (RCTs) assessing the effectiveness and safety of EPSW for the treatment of PSSHS in the following databases from their start to February 1, 2020 without language and publication date limitations: Cochrane Library, MEDLINE, EMBASE, CINAHL, Web of Science, PsycINFO, Chinese Biomedical Literature Database, and China National Knowledge Infrastructure. For trials that meet all inclusion criteria, 2 researchers will independently extract the data from them and appraise study quality by Cochrane risk of bias. Any differences will be solved by discussion with the help of another researcher. All data will be performed and analyzed using RevMan 5.3 software.

**Results:**

We will summarize up-to-date high quality RCTs to evaluate the effectiveness and safety of EPSW for the treatment of PSSHS.

**Conclusions:**

This study will provide a comprehensive evidence summary to determine whether EPSW is effective and safety for the treatment of PSSHS or not.

**PROSPERO registration number:**

PROSPERO CRD42020175630.

## 1 Introduction

Post-stroke shoulder-hand syndrome (PSSHS) is a common sequela of patients with stroke.^[1–4]^ Its mainly symptoms include hemiplegic shoulder pain, hyperalgesia, joint swelling, and limitations while moving.^[5–7]^ It has been estimated that its prevalence varies from 12% to 49%, and its incidence is about 70%.^[8–10]^ Although its treatment strategy approach has made great progress, its pathogenesis still remains unclear.^[11,12]^

Currently, clinical trials have reported extracorporeal shock wave (EPSW) for the treatment of PSSHS,^[13–18]^ but the effectiveness and safety has not been proved by systematic review. Therefore, this study aims to assess the effectiveness and safety of EPSW as a clinical management for patients with PSSHS.

## 2 Methods

### 2.1 Study registration

This protocol has been registered at PROSPERO (CRD42020175630). This study will follow the guidelines of the Preferred Reporting Items for Systematic Reviews and Meta-Analysis Protocol statement.^[19,20]^

### 2.2 Eligibility criteria

#### 2.2.1 Types of studies

Only randomized controlled trials (RCTs) which investigate the effectiveness and safety of EPSW for the treatment of PSSHS will be included. Animal studies, reviews, comments, case studies, non-clinical trials, uncontrolled clinical trials, non-RCTs, and quasi-RCTs will all be excluded.

#### 2.2.2 Types of interventions

We will only include trials which used EPSW as solely treatments for managing patients with PSSHS.

The control intervention could be any management. However, we will exclude studies that used any forms of EPSW as their control therapy, including EPSW combined with other treatments.

#### 2.2.3 Types of participants

Participants who were diagnosed as PSSHS will be included in this study. There will be no limitations to the race, sex, age, severity and duration of PSSHS, and economic status.

#### 2.2.4 Types of outcome measurements

Primary outcome is motor function of upper limbs, as measured by Fugl-Meyer Assessment scale or any other relevant scales.

Secondary outcomes are pain intensity (as assessed by visual analog scale or other pain tools), quality of life (as evaluated by Barthel Index or any other related indexes), severity of PSSHS (as checked by Shoulder Hand Syndrome Scale or other associated scores), and adverse events.

### 2.3 Search strategy

#### 2.3.1 Electronic databases sources

We will comprehensively search relevant RCTs in the following electronic databases from their beginning to the February 1, 2020 regardless language and publication date: Cochrane Library, MEDLINE, EMBASE, CINAHL, Web of Science, PsycINFO, Chinese Biomedical Literature Database, and China National Knowledge Infrastructure. The search strategy with details of Cochrane Library is created (Table 1). Similar search strategies of other electronic databases will be built.

**Table 1:**
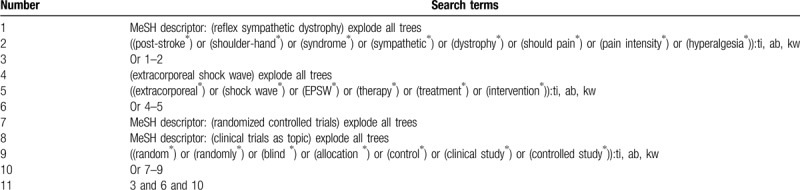
Search strategy utilized in Cochrane Library.

#### 2.3.2 Other literature sources

Besides the electronic databases, we will inspect other literature sources, such as dissertations, ongoing trials, conference proceedings, and reference lists of included trials.

### 2.4 Study selection

**Figure 1:**
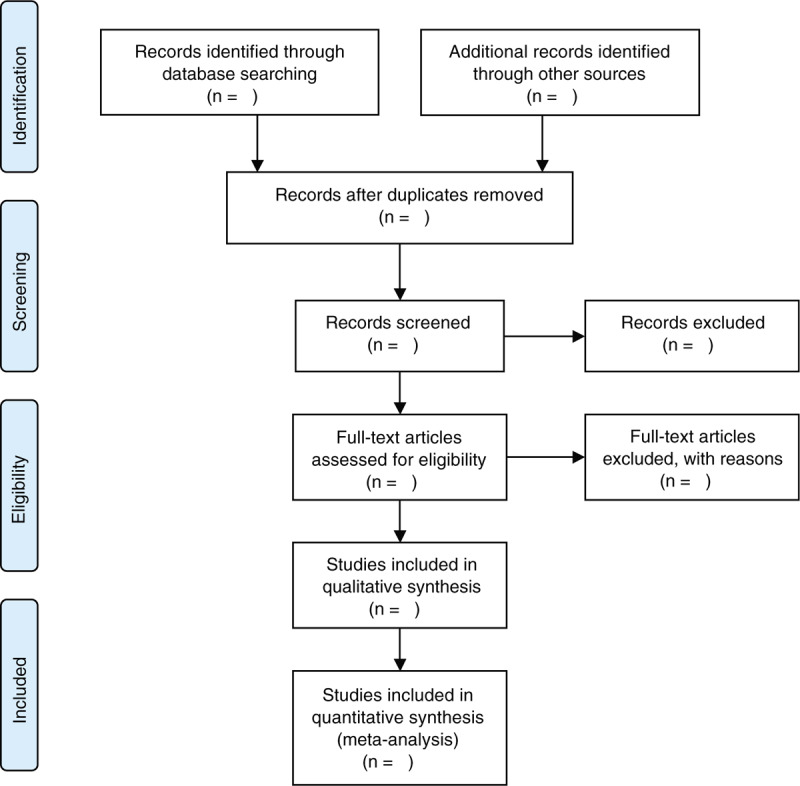
Flow chart of study selection process.

We will use EndNote X9 software (Clarivate Analytics, Philadelphia, USA) to manage all searched results, and any duplicates will be removed. Two researchers will review the titles/abstracts of each identified record independently to take away studies that do not fulfill eligibility criteria. According to the preliminary selection results, a full-manuscript investigation will be performed against all inclusion criteria. If there are any divergences between 2 researchers, they will be solved by discussion with another researcher. All excluded studies will be recorded with specific reasons and will be listed in the table. The details of whole study selection procedure will be presented in the flowchart (Fig. 1).

### 2.5 Data extraction

Based on the eligibility criteria, a standard data collection sheet containing specified outcome indicators will be created before data extraction. For trials fulfilling the inclusion criteria, 2 investigators will independently extract data from each eligible trial. If any differences occur between 2 investigators, they will be resolved by discussion with another investigator. The extracted information consists of general information (such as first author, country, time of publication, etc.), participant characteristics, study design (such as randomization method, blind, etc.), trial setting, specifics in the treatment and control groups (such as dosage, frequency, etc.), and outcome indicators, safety, and any other relevant information.

### 2.6 Assessment of risk of bias

Two investigators will independently assess the study quality using Cochrane risk of bias tool, which includes 7 aspects and each one is further judged as low, unclear, or high risk of bias. Any different opinions will be solved through discussion, or if they still cannot be reached, a third investigator will be consulted to reach a consensus.

### 2.7 Missing data management

If we identify incomplete or missing data during the period of data extraction, we will connect primary authors to request it. If we cannot receive those data, we will only analyze available data using intention-to-treat analysis.

### 2.8 Measurements of treatment effect

All continuous data will be estimated by mean difference or standardized mean difference and 95% confidence intervals (CIs), while all dichotomous data will be calculated as risk ratio and 95% CIs.

### 2.9 Assessment of heterogeneity

We will examine statistical heterogeneity across include trials using *I*^*2*^ test. *I*^2^ ≤ 50% means homogeneity, and a fixed-effects model will be utilized. *I*^2^ > 50% means considerable heterogeneity, and a random-effects model will be employed.

### 2.10 Subgroup analysis

A subgroup analysis will be carried out to explore the sources of the obvious heterogeneity according to the difference in study characteristics, treatments, controls and outcome indicators.

### 2.11 Sensitivity analysis

A sensitivity analysis will be undertaken to test the stability and robustness of study findings according to the impacts of study quality, sample size, and missing data.

### 2.12 Reporting bias

A funnel plot and Egger regression test will be performed to find out if any reporting biases exist when at least 10 RCTs are included.^[21,22]^

### 2.13 Data synthesis

Statistical analysis will be conducted using RevMan 5.3 software (Cochrane Community, London, UK). When there is homogeneity across >2 included trials, we will perform a meta-analysis according to similarity of study characteristics, interventions, controls, and outcome measurements. When there is obvious heterogeneity, we will carry out subgroup analysis. If it is impossible to undertake a meta-analysis, we will report a qualitative discussion and a narrative summary for the outcome results.

## 3 Discussion

PSSHS is a serious threat to the quality of life in patients with stroke. Although EPSW is reported to treat PSSHS effectively, all conclusions are drawn based on the individual study. In addition, there is no systematic review of the clinical evidence of EPSW treatment for PSSHS up to now. Thus, this study intends to carry out a systematic review to assess the effectiveness and safety of EPSW in the treatment of PSSHS. We hope this study can provide evidence and options to clinicians for the treatment of PSSHS.

### 3.1 Ethics and dissemination

This study is based on the published studies; therefore, no ethical approval is inquired. The results of this study are expected to be published at a peer-reviewed journal.

## Author contributions

**Conceptualization:** Tian-shu Wang, Yu Zhao.

**Data curation:** Tian-shu Wang, Zhao-chen Tang.

**Formal analysis:** Tian-shu Wang, Wei-dong Song, Zhao-chen Tang.

**Investigation:** Yu Zhao.

**Methodology:** Wei-dong Song, Zhao-chen Tang.

**Project administration:** Yu Zhao.

**Resources:** Tian-shu Wang, Shou-feng Wang, Wei-dong Song, Zhao-chen Tang.

**Software:** Tian-shu Wang, Shou-feng Wang, Wei-dong Song, Zhao-chen Tang.

**Supervision:** Yu Zhao.

**Validation:** Tian-shu Wang, Shou-feng Wang, Wei-dong Song, Yu Zhao, Ken Lee.

**Visualization:** Tian-shu Wang, Shou-feng Wang, Wei-dong Song, Zhao-chen Tang, Yu Zhao, Ken Lee.

**Writing – original draft:** Tian-shu Wang, Shou-feng Wang, Wei-dong Song, Zhao-chen Tang, Yu Zhao.

**Writing – review & editing:** Tian-shu Wang, Wei-dong Song, Zhao-chen Tang, Yu Zhao, Ken Lee.
